# Effective inhaler technique education is achievable - assessment and comparison of five inhaler devices errors

**DOI:** 10.3389/fphar.2025.1538283

**Published:** 2025-04-24

**Authors:** Monika Marko, Magdalena Klimczak, Marharyta Sobczak, Maciej Wojakiewicz, Tomasz Dębowski, Andrzej Emeryk, Rafał Pawliczak

**Affiliations:** ^1^ Department of Immunopathology, Faculty of Medicine, Division of Biomedical Science, Medical University of Lodz, Lodz, Poland; ^2^ Department of Pulmonary Rehabilitation, Lung Diseases Treatment and Rehabilitation Center in Lodz, Lodz, Poland; ^3^ Medical Department, Chiesi Poland Sp. z o.o., Warsaw, Poland; ^4^ Department of Pulmonary Diseases and Children Rheumatology, Medical University of Lublin, Lublin, Poland; ^5^ Department of Pediatrics Pulmonology and Rheumatology, Pediatrics University Hospital in Lublin, Lublin, Poland

**Keywords:** asthma, devices, education, errors, inhalation therapy, inhaler, technique

## Abstract

**Objective:**

The study is based on a respiratory educational program aimed at training medical personnel to use inhalers correctly and educating patients on improving their inhalation skills.

**Methods:**

Adult patients with asthma were divided into groups according to the inhaler: Ellipta, Diskus, Cyclohaler, Pressurized metered-dose inhaler (pMDI), and Turbuhaler. Patients were assessed for inhalation skills and then educated by previously trained nurses. The results were collected in forms allowing the evaluation of the number of critical and other errors made by patients.

**Results:**

The number of errors during inhalers use decreased at subsequent visits after education. The number of critical errors was lower than other errors for each device before and after education. Statistically significant differences in the inhalation technique assessment (before education, visit 1, and visit 2) were shown for Cyclohaler and pMDI (p < 0.0001), Turbuhaler (p = 0.0014), Diskus (p = 0.0025) and Ellipta (p = 0.0091).

**Conclusion:**

Before education, the least technical difficulties were observed for the Cyclohaler, while in the Ellipta group, patients made the most errors. Education resulted in equalizing the level of correctness of inhalation, which was similarly high for each type of device. This means that after education, all inhalers have similar difficulty levels in performing the correct inhalation technique. However, achieved improvement may be influenced by other factors such as practice effects or confounding variables due to real-life nature of the study.

## 1 Introduction

Respiratory diseases, such as chronic obstructive pulmonary disease (COPD) and asthma, are associated with high morbidity and mortality and, therefore, constitute a severe global health problem (Cataldo et al., 2022). The mainstay of treatment for these diseases is inhaled therapy (van der Palen et al., 2016; Bosnic-Anticevich et al., 2023). The advantage of such an approach is the rapid and targeted drug delivery to the lungs with limited potential side effects and systemic drug exposure (Cataldo et al., 2022). Different types of inhalation devices are available: pressurized metered dose inhalers (pMDI), dry-powder inhalers (DPI), soft-mist inhalers, and nebulizers (Cataldo et al., 2022; Sorino et al., 2020; Arnold et al., 2022). In theory, it may seem that the use of inhalers is straightforward. However, in practice, it turns out that only a small proportion of patients use the inhaler correctly, which is the main reason for the ineffective treatment of asthma and COPD. The most important are critical errors that result in no drug delivery in the lungs (Cataldo et al., 2022; Usmani et al., 2018). Moreover, individual, patient-related factors may alter the inhaler’s effectiveness (Bosnic-Anticevich et al., 2023). According to the Global Initiative for Chronic Obstructive Lung Disease (GOLD) Report (Global Initiative for Chronic, 2024), over two-thirds of patients make at least one mistake when using their inhaler. The problem of incorrect use of inhalers has been analyzed among adolescents and children (Frey et al., 2023; Kimel, 2022). It has been shown that long-term use of pharmacotherapy is crucial in the treatment of chronic diseases such as asthma, yet only half of children take their medications as prescribed (Frey et al., 2023). Many children and adolescents have been shown not to use their inhalers correctly. The role of school nurses in assessing inhaler technique and providing knowledge about inhaler use is also emphasized (Kimel, 2022).

Moreover, it was demonstrated that no inhaler eliminates errors and the need to explain, demonstrate or regularly review inhalation techniques (Bosnic-Anticevich et al., 2023; Global Initiative for Chronic, 2024). Unfortunately, leaflets attached to product packaging are insufficient to educate patients about the inhaler use (Global Initiative for Chronic, 2024). Other strategies, such as physical training and video or web-based education, improved the inhaler technique in some patients, but the effects diminished over time (Klijn et al., 2017). On the other hand, the “teach-back” method, when patients should show how to use the device, seems to be especially effective (Global Initiative for Chronic, 2024). A similar problem with the use of inhalers exists in asthma patients–up to 70%–80% do not use the inhaler correctly, according to the Global Initiative for Asthma (GINA) Main Report (GINA, 2023). Similarly to the GOLD Report (Global Initiative for Chronic, 2024), the GINA Main Report states that physical demonstration and regular revisions are necessary for perfecting the inhalation technique in asthmatic patients, especially in patients with poor asthma control. Of note, routine correcting the inhaler use takes merely 2–3 min and significantly improves asthma control in older children and adults (GINA, 2023). Currently, more than educating patients on the correct inhalation technique is needed. Adequate communication at the medical personnel-patient level must also be improved (Plaza et al., 2018). This, in turn, may still result in doctors or nurses needing to be more adequately qualified to work with a patient using an inhaler as therapy (Plaza et al., 2018; Xie et al., 2023). Attention is also drawn to the need for repetition and reminding of training (Barnestein-Fonseca et al., 2023; Román-Rodríguez et al., 2019). Many studies conducted so far also emphasize the role of the pharmacist in guiding the patient in the proper use of inhalation devices (Gul et al., 2023; Hussain et al., 2025; Ghozali and Mutiara, 2024). Various educational methods, such as mobile applications, have been developed to facilitate the understanding of inhaler use by pharmacists and healthcare personnel (Ghozali and Mutiara, 2024). The views and experiences of asthma patients and pharmacists regarding the use of inhalers were also examined (Hussain et al., 2025). Pharmacists have been shown to play a key role in supporting patients by counseling them on the use of inhalers (Gul et al., 2023).

Considering the relevance of education and training in inhalation techniques, we decided to carry out an educational project to spread awareness and encourage proper inhalation skills among asthma patients and medical personnel. The project aimed to train nurses from health center and hospital pulmonology departments - to properly educate patients during visits. An additional purpose of the study was to compare the number of errors in the inhalation technique made by patients before and after education, depending on the inhalation device used. It should be emphasized that although patient education improves inhalation techniques, the problem of incorrect use of inhalers still exists. Many patients and medical personnel still need to correct their use of inhalers, which indicates deficiencies in existing recommendations and educational approaches. Therefore, it was assumed that the results of this educational project would provide essential data and contribute to the formulation of recommendations for educating medical personnel and patients on the use of inhalers.

The key difference that sets this study apart from currently available research is the emphasis on training nurses, who then pass on the knowledge to patients. This approach offers important benefits for better patient education and improved patient outcomes. Nurses often have direct contact with patients over a more extended period. Thanks to regular interactions, they can better recognize patients’ difficulties in using inhalers and identify factors that may affect the quality of treatment. Compared to a single training conducted by a physician, this approach allows for better adaptation of education to the specific needs of patients. The study differs from the previously described studies in that its main idea was to conduct an educational program for nurses and patients and then check how the education affects the correctness of the inhalation technique among patients using various devices. Many results from educational programs designed exclusively for this purpose have yet to be published. The results of studies were described, aiming to check a specific type of education, e.g., video education, face-to-face training, brief education or puzzle game, in improving the inhalation technique (Kan and Şen, 2022; Carpenter et al., 2016; Kellman et al., 2020). Also, in the literature are descriptions of research comparing two educational methods, e.g., Virtual Teach-to-Goal (V-TTG) and brief intervention (Volerman et al., 2020a). Descriptions of research findings on factors associated with inappropriate use of inhalers before and after education are also available (Trela et al., 2022). However, the issue of achieving education itself was not discussed individually. It was not checked or discussed which type of inhalation device education is possible and to what extent. In our study, we focused on achieving the highest level of education for nurses and patients and improving inhalation techniques, which enabled us to analyze the problem closely. The study’s strength is that one specialist carried out the education within a specific time frame at a meeting with nurses organized for this purpose, guaranteeing the consistency of the knowledge provided on the correct inhalation technique.

## 2 Materials and methods

Adults with asthma were eligible for enrolment. Patients were divided into five groups according to the type of inhaler used: Ellipta, Diskus, Cyclohaler, pMDI, and Turbuhaler. The project was carried out in three steps: 1) recruitment of nurses working with patients with lung diseases, 2) practical training in inhalation techniques for nurses from the health center and hospital pulmonology departments, and 3) individual nurse-patient meetings (an interview, patient education and inhalation technique assessment). The practical training for nurses included the following substantive and practical parts (learning the inhalation technique). Then, the trained skills were verified during exercises in pairs, discussing mistakes made and presenting the correct inhalation technique to standardize the nurses’ level of inhalation skills. This part of the educational program was also intended to adequately prepare nurses for patient training and consistently assess patients’ inhalation techniques. The nurses’ training, practical exercises and verification of their skills were conducted by one specialist in the field of respiratory diseases.

The part of the project involving patients was also divided into stages. Stage I included verification of the inhalation technique and marking on the inhalation technique assessment scale (baseline, before education), education, remarking on the inhalation technique assessment scale by the nurse (visit 1), and providing education materials. Stage II included verification of the inhalation technique, marking the inhalation technique on the scale and re-education (visit 2). Specially developed forms were used to examine patients’ skills in using inhalers. Although there are many assessment tools related to inhalation, none have been fully validated for a comprehensive assessment of the inhaler technique. Therefore, to adapt the methodology to the specificity of the problem under study, we developed our own assessment questionnaire, considering all aspects of correct inhaler use that would be appropriate for our study group and the purpose of the study. The questionnaire was based on officially approved “Patient Information Leaflets” for particular medicinal products/inhalers. The nurse completed the forms before the education, immediately after (at Visit 1), and during the following visit (at Visit 2). The forms were developed to determine the current patients’ inhalation technique and, therefore, the number of errors made by patients when using five different inhalers. A questionnaire was developed for each device in the form of a checklist of correctly performed maneuvers. Nurses marked in the questionnaire correctly and incorrectly performed maneuvers by patients before and after education. Nurses completed the questionnaires in real-time during the meeting with the patient and the patient’s inhalation procedure. Questionnaires prepared this way ensured consistency in nurses’ assessment of the inhalation technique. Among the errors that could be made for each inhaler, we listed two types of errors: critical and noncritical errors. Critical errors could significantly impede the delivery of the appropriate drug to the lungs. “Noncritical” (other) errors include those that are likely to result in less drug reaching the lungs compared to the amount obtained using the correct technique (Usmani et al., 2018). The list of items assessed in the questionnaire for each inhaler used in the study was summarized in Supplementary Table S1, detailing critical and noncritical errors. The function of the questionnaire was only to count errors. It was not decided to conduct validation because the study was only aimed at monitoring errors in the use of the inhaler in a very general scope, and the data will not be used for complex statistical analyses or inferences about the causes of errors. Furthermore, because, in our case, the data analysis was performed by one rater for one patient, the inter-rater test has not been applied. Since the study was educational and pilot in nature, it was conducted on a small group of patients. This allows for a preliminary assessment of the questionnaire’s operation and may help identify possible difficulties in using the tool before its full implementation. Comparison between the inhalers could be performed, even though they have different activation mechanisms, utilizing the same assessment scoring. Some patients, despite the European Respiratory Society (ERS) (Papi et al., 2023), European Academy of Allergy and Clinical Immunology (EAACI) (Mathioudakis et al., 2021) and Global Initiative for Asthma (GINA) (GINA, 2023) guidelines and statements, use more than one type of inhaler, therefore universal scoring underlines the possibility of comparison various inhalers in a typical clinical setting.

To ensure data integrity, each person involved in the study had to be familiar with the study protocol. The study protocol specified how nurses should assess the inhalation technique and what errors should be recorded. Assessment criteria that are easy to apply in practice were used (marking incorrect and correct maneuvers). A clear and intuitive form was designed to easily record observations. The form included verification fields (selection from a list of predefined errors) to reduce the risk of recording errors. The collected data was checked for compliance with the protocol. Data collected by different nurses were compared to detect any discrepancies in the assessments. Nurses had the opportunity to report doubts regarding the assessment of inhalation technique or classification of errors, which allowed them to avoid subjectivity and ensure consistency of assessments. Demographic data were collected at baseline and were also recorded in the questionnaires.

This is a prospective, observational, non-interventional study conducted in real-world conditions. The study protocol was subject to routine clinical practice but did not dictate the allocation of patients to the study group. The physician decides the treatment choice based on clinical needs under the relevant guidelines and authorizations for the medicinal product. Enrollment of patients in the study was separate from the decision to prescribe the drug. No additional diagnostic procedures or monitoring of vital parameters were performed on the patients. Accordingly, the study meets the criteria of a “non-interventional trial” specified in Directive 2001/20/EC–Article 2(c) (“*a study where the medicinal product(s) is (are) prescribed in the usual manner in accordance with the terms of the marketing authorisation. The assignment of the patient to a particular therapeutic strategy is not decided in advance by a trial protocol but falls within current practice and the prescription of the medicine is clearly separated from the decision to include the patient in the study. No additional diagnostic or monitoring procedures shall be applied to the patients and epidemiological methods shall be used for the analysis of collected data*”) (European Parliament and of the Council, 2024). The study meets the definition of a non-interventional study because did not assess the impact of education on biomedical or health-related outcomes. Only improved inhaler technique was assessed, but no further health consequences related to the level of inhaler technique were examined. Furthermore, due to the real-world nature of the study, a set protocol was not followed, and participants used their inhalers during routine medical care. Education on inhaler technique is not an intervention because its primary goal is to improve the patient’s inhalation skills rather than to introduce a new, active agent that directly changes health status, as is the case with administering of an intervention such as a drug. In this study, education aimed to provide the patient with knowledge and instructions on using the inhaler correctly, allowing them to manage their therapy better. Such interventions are supportive and complementary but are not interventions in themselves because they do not involve the introduction of a substance that directly affects the biological mechanisms (does not change the disease mechanism itself).

Given the above criteria, the study design and methodology were developed following the provisions for non-interventional trials in the Polish Pharmaceutical Law Act (Polish Pharmaceutical Act, 2024). According to the Art.37al. (Polish Pharmaceutical Act, 2024) of the Polish Pharmaceutical Law Act, the provisions concerning the need to obtain the consent of the Local Ethics Committee (Polish Pharmaceutical Law Act, Chapter 2a “Clinical trials of medicinal products”), do not apply to non-interventional studies. Consequently, according to Polish law, the Local Ethics Committee does not have to approve the study protocol. This is a purely observational study, not interfering with treatment. Therefore, an informed consent is also not required (Pharmaceutical Law Act, Art.37al.) (Polish Pharmaceutical Act, 2024).

### 2.1 Statistical analysis

Statistical analysis was performed using GraphPad Prism 10.2.0 software. When the variables were normally distributed, determined by the Shapiro–Wilk test, the differences between the three groups were assessed by the Brown-Forsythe ANOVA test with Dunnett’s multiple comparison test. When the variables were not normally distributed, the differences between the three groups were assessed by the Friedman ANOVA test with Dunn’s multiple comparison test. The differences between the two groups were assessed by a paired t-test, in which the distribution of variables was in accordance with normality. When the variables did not pass the normality test, the Wilcoxon matched-pairs single rank test assessed the differences between two groups. The differences between the number of critical errors (I and II) and other errors were analyzed using the Chi-square test. The results were considered statistically significant at P < 0.05.

## 3 Results

### 3.1 Background and baseline characteristics of the study population

The study was conducted between 24 November 2022 and 5 April 2023 in Poland and included 89 patients and 13 nurses from the health center and hospital pulmonology departments. A summary of patients’ characteristics at baseline is shown in Table 1. The average number of days between visits one and two was 18.27 ± 5.04 (mean ± SD). The score was established as a percentage of correctly performed maneuvers to all maneuvers, particularly the inhaler check list form (Supplementary Table S1). At baseline (before education), the score in the assessment of inhalation technique achieved by patients was as follows: in the Ellipta group (55.29 ± 9.896), in Cyclohaler group (84.00, 68.00; 89.00), (median with lower and upper quartiles), in pMDI group (75.00, 67.0; 83.00), in Diskus group (72.00, 57.25; 91.00), and Turbuhaler group (71.00, 57.00; 86.00) (Table 2). The most common mistake in the inhalation technique before education was shaking the inhaler in Cyclohaler (70%), Turbuhaler (57.14%), Diskus (83.33%), and Ellipta (100%) groups. This maneuver was considered incorrect because these inhalers do not require shaking. However, the most common mistake in the pMDI group (48.48%) was incorrect exhalation when using the device. The average score before education was obtained for Cyclohaler (76%), Turbuhaler (73%), Diskus (70%), and Ellipta (55%), respectively (Supplementary Table S2).

**TABLE 1:** Summary of patient characteristics.

	Ellipta (N = 7)	Diskus (N = 12)	Cyclohaler (N = 30)	pMDI (N = 33)	Turbuhaler (N = 7)	Total (N = 89)
Gender, n (%)						
Male	5 (71.43)	2 (16.67)	14 (46.67)	9 (27.27)	1 (14.29)	31 (34.83)
Female	2 (28.57)	10 (83.33)	16 (53.53)	24 (72.73)	6 (85.71)	58 (65.17)
Age, years, mean (SD)	72.57 (8.08)	71 (10.71)	68.9 (10.63)	61.82 (12.95)	71 (11.39)	67.01 (11.96)
Asthma history, years n (%)						
<1	0	1 (8.33)	0	2 (6.06)	1 (14.29)	4 (4.49)
≥1 to <5	3 (42.86)	5 (41.67)	6 (20.00)	7 (21.21)	0	21 (23.60)
≥5 to <15	1 (14.29)	4 (33.33)	13 (43.33)	18 (54.55)	3 (42.86)	39 (43.82)
≥15	3 (42.86)	2 (16.67)	11 (36.67)	6 (18.18)	3 (42.86)	25 (28.09)
Inhalation technique education in the past n (%):						
Yes No	4 (57.14) 3 (42.86)	6 (50.00) 6 (50.00)	20 (66.67) 10 (33.33)	21 (63.64) 12 (36.36)	4 (57.14) 3 (42.86)	55 (61.8) 34 (38.2)

Abbreviations: pMDI, pressurized metered-dose inhaler; SD, standard deviation.

**TABLE 2:** Inhalation technique assessment scale results (total score expressed as a percentage).

	Before education	Visit 1	Visit 2	ANOVA^a^ *p*
Ellipta	55.29 ± 9.896	78.71 ± 7.486	94.00 ± 2.380	0.0091
Diskus	72.00 (57.25; 91.00)	91.00 (84.25; 97.75)	100.00 (91.00; 100.00)	0.0025
Cyclohaler	84.00 (68.00; 89.00)	95.00 (95.00; 95.00)	95.00 (95.00; 100)	<0.0001
pMDI	75.00 (67.00; 83.00)	100.00 (100.00; 100.00)	100.00 (100.00; 100.00)	<0.0001
Turbuhaler	71.00 (57.00; 86.00)	100.00 (93.00; 100.00)	100.00 (93.00; 100.00)	0.0014

Abbreviations: pMDI, pressurized metered-dose inhaler.

^a^ For Ellipta, the distribution of variables was in accordance with normality determined by the Shapiro–Wilk test, and the differences between the three groups were assessed by the Brown-Forsythe ANOVA, test. The obtained results were averaged and presented as mean ± standard error of the mean (Mean ± SEM). For Diskus, Cyclohaler, pMDI, and Turbuhaler, the distribution of variables was not in accordance with normality determined by the Shapiro–Wilk test; the differences between the three groups were assessed by the Friedman ANOVA, test. The obtained results are presented as medians with lower and upper quartiles.

### 3.2 Findings over the subsequent study visits

**FIGURE 1:**
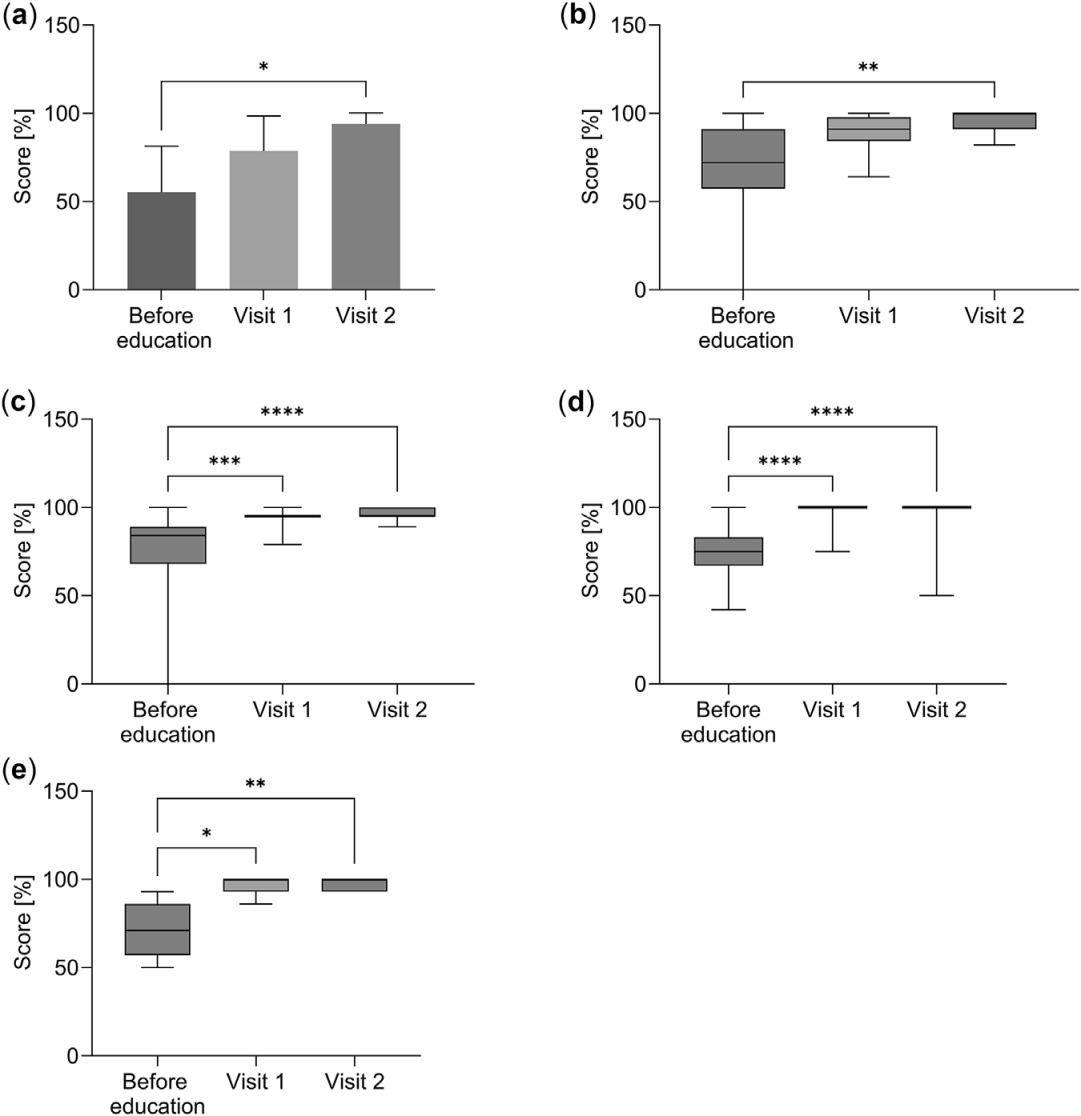
Inhalation technique assessment scale results (total score expressed as a percentage). **(a)** Ellipta. **(b)** Discus. **(c)** Cyclohaler. **(d)** Pressurized MDI. **(e)** Turbuhaler. For Ellipta the results were averaged and presented as mean ± standard error of the mean (Mean ± SEM). For Discus, Cyclohaler, Pressurized MDI and Turbuhaler. The results are presented as medians with lower and upper quartiles. For Ellipta, the differences between the three groups were assessed by the Brown-Forsythe ANOVA test with Dunnett’s multiple comparison test. For Diskus, Cyclohaler, pMDI, and Turbuhaler, the differences between the three groups were assessed by the Friedman ANOVA test with Dunn’s multiple comparison test. Statistically significant differences were found at the level of *p < 0.05, **p < 0.01, ***p < 0.001, ****p < 0.0001.

The study showed a statistically significant increase in the inhalation technique assessment scale at subsequent visits (Table 2). The total score expressed as a percentage increased during each subsequent study visit in each study group. Statistically significant differences between three groups (before education, visit one and visit 2) were shown for Cyclohaler and pMDI (p < 0.0001), Turbuhaler (p = 0.0014), Diskus (p = 0.0025) and Ellipta (p = 0.0091) respectively (Table 2). Statistically significant differences between groups (multiple comparisons) – before education and Visit two were found for Ellipta (p < 0.05), (Figure 1a), and Discus (p < 0.01), (Figure 1b). Statistically significant differences between baseline and Visit one and baseline and Visit two were found for Cyclohaler (baseline vs Visit one p < 0.001 and baseline vs Visit two p < 0.0001), (Figure 1c), pMDI (baseline vs Visit one p < 0.0001 and baseline vs Visit two p < 0.0001), (Figure 1d), and Turbuhaler (baseline vs Visit one p < 0.05 and baseline vs Visit two p < 0.01), (Figure 1E). Results from multiple comparisons indicate that for each inhaler assessed, there was a statistically significant improvement in inhalation technique before education compared to visit 2.

The summary of the results showed that the average score improvement (a percentage of correctly performed maneuvers to all maneuvers) at the first visit was 23% in the Ellipta group, 19% in the Diskus group, 18% in the Cyclohaler group, 21% in the pMDI group and 22% in the Turbuhaler group. The average difference in score between Visit one and Visit two was in the Ellipta (15%), Diskus (7%), Cyclohaler (2%), pMDI (1%) and Turbuhaler groups (1%). It also should be emphasized that average results after Visit one were 79% in the Ellipta group, 89% in Diskus, 94% in Cyclohaler, 97% in pMDI, and 96% in Turbuhaler groups. The average score after education was similar for all inhalers (94%–99%), indicating the education’s effectiveness (Supplementary Table S2).

**FIGURE 2:**
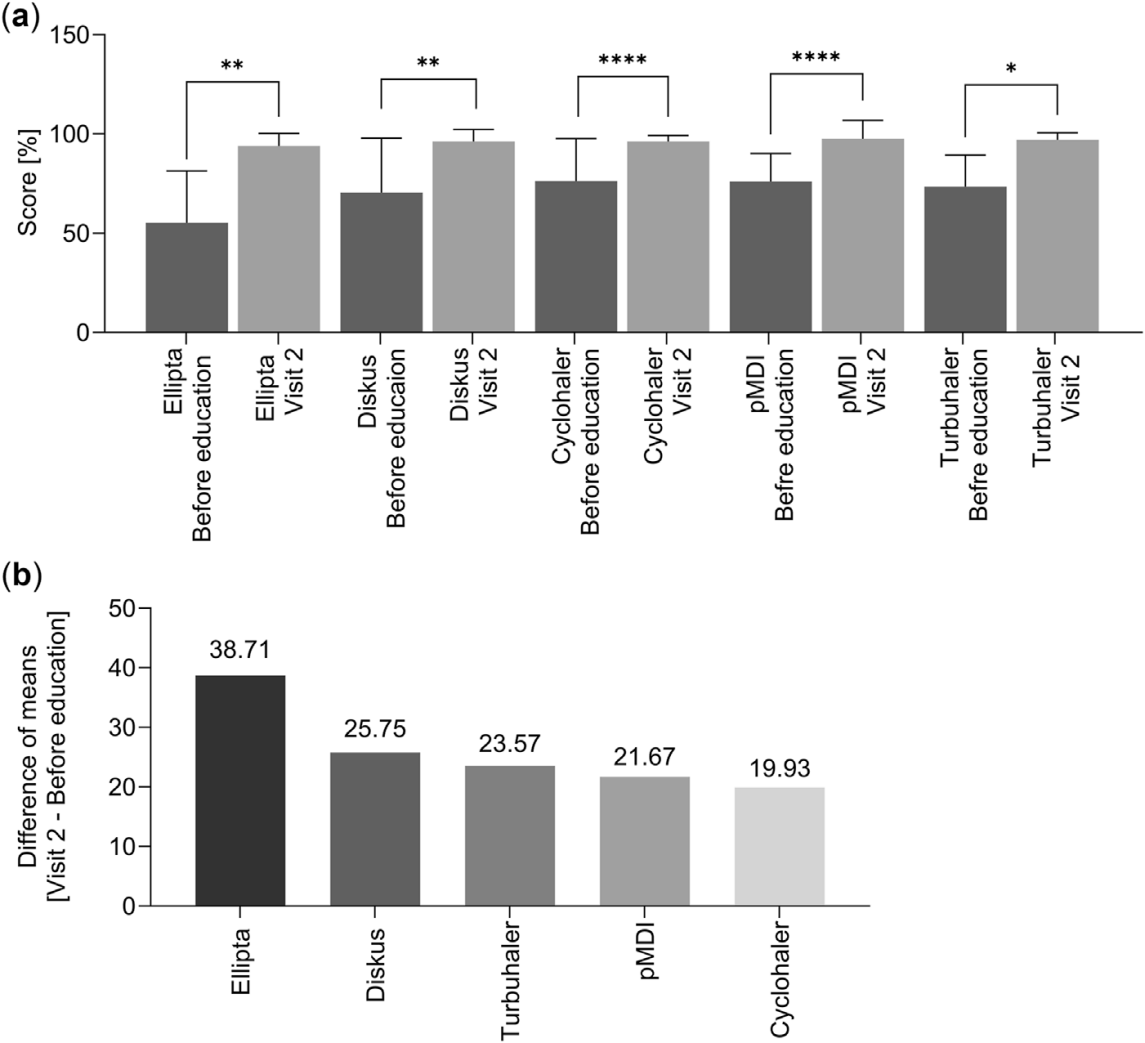
Differences in obtained score. **(a)** Differences in obtained score before education and Visit 2. The results were averaged and presented as mean ± standard error of the mean (Mean ± SEM). Statistically significant differences were found at the level of *p < 0.05, **p < 0.01, ****p < 0.0001. **(b)** Difference of means of score from Visit two and means of score obtained before education.

The study showed statistically significant differences in obtained scores before education and Visit two for Ellipta and Diskus (p < 0.01), Cyclohaler and pMDI (p < 0.0001), and Turbuhaler (p < 0.05) (Figure 2a; Table 3). Additionally, the difference in means of score from Visit two and the means of score obtained before education were compared (Figure 2b). Figure 2a shows that after education, patients in all groups achieved similar results in improving their inhalation technique. This means that all inhalers have similar difficulty learning to perform the correct inhalation technique after education. In Figure 2b, the lowest score (Cyclohaler) means that there was the slightest difference in progress in improving the inhalation technique between visit two and the inhalation before education. This means that Cyclohaler was the most intelligible for the patient before education. The highest score (Ellipta) means there was the most remarkable difference in progress in improving the inhalation technique between visit two and the inhalation before education. This means that Ellipta was the least intelligible for the patient before education. However, education contributed to achieving the highest progress in improving the inhalation technique in this group.

**TABLE 3:** Differences in obtained score before education and Visit 2.

	Score before education [mean]	Score visit 2 [mean]	Mean of differences visit 2 vs. Before education	*p*
Ellipta^a^	55.29	94.00	38.71 ± 7.87	0.0027
Diskus^b^	70.5	96.25	25.75; 23.50	0.0059
Cyclohaler^b^	76.33	96.27	19.93; 16.00	<0.0001
pMDI^b^	76.06	97.73	21.67; 25.00	<0.0001
Turbuhaler^b^	73.43	97.00	23.57; 29.00	0.0156

Abbreviations: pMDI, pressurized metered-dose inhaler.

^a^ For Ellipta, the distribution of variables was in accordance with normality determined by the Shapiro–Wilk test; the differences between the two groups (Before education vs Visit 2) were assessed by the Paired t-test. The obtained results were averaged and presented as mean ± standard error of the mean (Mean ± SEM).

^b^ For Diskus, Cyclohaler, pMDI, and Turbuhaler, the distribution of variables was not in accordance with normality determined by the Shapiro–Wilk test. The differences between the two groups (Before education vs Visit 2) were assessed by the Wilcoxon matched-pairs single-rank test. The results are presented as mean differences between the two groups and median (Mean; median).

The number of errors made by patients during inhalation decreased during subsequent visits after education. The analysis showed that the number of critical errors was lower than others for each device before and after education. Chi-square test showed statistically significant differences between the number of critical errors (I and II) and other errors for Ellipta: other errors before education to Visit one (p = 0.0005) and before education to Visit two (p < 0.0001), critical error II before education to Visit two (p = 0.005); for Diskus: other errors before education to Visit one (p = 0.0002) and before education to Visit two (p < 0.0001); for Cyclohaler: critical error I before education to Visit one (p = 0.02) and Visit two (p = 0.02), other errors before education to Visit one (p < 0.0001) and before education to Visit two (p < 0.0001); for pMDI: other errors before education to Visit one (p < 0.0001) and before education to Visit two (p < 0.0001), critical error I before education to Visit two (p = 0.04); for Turbuhaler: other errors before education to Visit one (p < 0.0001) and before education to Visit two (p < 0.0001), (Supplementary Table S3).

Additionally, the number of errors made by patients when using each of the five inhalers during subsequent study visits was calculated. The proportional distribution of errors considered critical when using the inhaler in relation to other errors is shown in Supplementary Figures S1-S5.

## 4 Discussion

The analysis of the results obtained in this study showed that patients using five types of inhalers had difficulty with their correct use before education. However, after education patients achieved progress in improving inhalation techniques in all assessed groups. Because of the real-life nature of the study, observed improvement may be due to education but could also be influenced by other factors such as practice effects or confounding variables. Patients using Ellipta had the highest difficulty in correctly using the inhaler before education. On the other hand, education contributed to achieving the highest progress in improving inhalation techniques in this group. The most common mistake in the Ellipta, Diskus, Cyclohaler, and Turbuhaler groups was shaking the inhaler before use. This maneuver was considered an error because these devices do not require shaking before use. Meanwhile, patients using pMDI skipped the step of exhaling as slowly as possible out of the inhaler. It should also be emphasized that on visit 2, patients in all groups achieved a high, similar average score in assessing the correctness of inhalation. These results indicate that education was effective and possible for each of the five inhalers used in the study, which helped improve inhalation skills and equalize the level of patients’ inhalation technique.

We also considered the possibility of making critical errors when using inhalers. Our study showed that critical errors occurred less frequently than other errors, and their number also decreased with patient education. In the case of Diskus, Cyclohaler, and Turbuhaler, critical errors I and II were eliminated after the first attempt at education and did not appear on visits one and 2. In the case of pMDI, the critical error “remove the protective cap from the inhaler mouthpiece” was not present before education and did not appear at subsequent visits.

Similar studies have been previously published (van der Palen et al., 2016; Volerman et al., 2020a; Trela et al., 2022; Jahedi et al., 2017; Janežič et al., 2020; Dabrowska et al., 2019). Some of them describe observations that are different from ours. We showed that Ellipta had the highest degree of difficulty, while Palen et al. (van der Palen et al., 2016) observed that asthma patients performed best with this inhaler, made the fewest errors, and did not require instruction. On the other hand, the same study showed that the number of critical errors in all tested inhalers was lower than the number of other errors, which in turn was consistent with the results of our study. Similar to our findings, Jahedi et al. (Jahedi et al., 2017) showed that most patients did not use their inhalers correctly. Also, the study by Janezic et al. (Janežič et al., 2020) concluded that most patients make at least one error in the inhalation technique. In this study, most mistakes were made by patients using Diskus. In contrast, the fewest mistakes made by patients using Turbuhaler. However, it should be considered that Ellipta and Cyclohaler were not included in this study.

Significant results were presented by Aydemir (2015) in the study concerned DPI and pMDI. This study showed that patients using pMDI made more errors before training. The training contributed to improving the rate of correct usage of inhalers in both cases; however, in the pMDI group, this rate was lower compared to DPI. A noteworthy observation is that some patients in the pMDI group made the same mistakes even after training. Such events also occurred in several cases in our study. We showed that in each group, most patients demonstrated improved inhaler use skills after education. These results are consistent with the observations of Bosnic-Anticevich (Bosnic-Anticevich, 2017), who pointed out that almost every patient can be taught how to use the inhaler properly regardless of the device.

In the present study, we also collected data on asthma history, which showed that most of the study patients had been suffering from the disease for more than 5–15 years. Additionally, 61.8% of patients declared that they had been educated on the correct inhalation technique by a doctor or medical personnel. Concerning these patients, helpful information could be how many types of devices the patient has encountered. However, such data were not collected in this study because patients with a long history of asthma may be unable to name, identify and distinguish all the types of inhalers they have used over the years of their illness, especially if they are elderly. Therefore, such data would not be reliable.

It should be emphasized that more than half of the patients participating in the study had received education in the past. However, when they participated in our project, the patients still could not perform inhalation without making mistakes. Therefore, it can be concluded that the education they had previously received was not carried out correctly, and the patients’ skills were not verified and improved during subsequent education. Additionally, the poor results of patients on the first visit during our education project, even though they had been educated earlier, prove that not every education carried out is effective. This, in turn, justifies the need to introduce our educational program, which resulted in a statistically significant improvement in patients’ inhalation technique and, consequently, improved nurses’ qualifications in this area. This conclusion confirms previously published expert opinions that there is still a need for research in the field of patient education and improvement of inhalation techniques (Bosnic-Anticevich, 2017; Volerman et al., 2020b; Bellanti and Settipane, 2016). The reason for incorrect inhalation technique may be the type of device, the patient’s lack of knowledge, or the healthcare provider (Bosnic-Anticevich, 2017; Price et al., 2013). Price et al. (Price et al., 2013) also described that a small percentage of patients receive inhaler education or inhaler technique consultation over time. As a result, approximately half of patients who initially learn to use their inhalers correctly do not maintain this correct technique over time. This phenomenon may explain the poor results in our study’s inhalation technique (before education) obtained by patients who have had asthma for many years. In this case, reference can also be made to the systematic review by Klijn et al. (Klijn et al., 2017), who concluded that educational interventions to improve inhalation technique are effective in the short term. This may also be the reason for poor inhalation techniques in patients with long asthma histories who were educated in our project.

Not only the lack of patient skills but also the quality of instructions given to patients by medical personnel must be a critical factor that can be improved to reduce inhaler mishandling (Klijn et al., 2017; Plaza et al., 2018). Based on the results obtained, patient education was effective, but the role of prior nurse training should also be emphasized. Patients improved their skills in the inhalation technique because they were instructed adequately by nurses who were properly prepared to conduct the training. This study shows a strong relationship between the importance of high qualifications of medical personnel in inhalation technique and achieving satisfactory results by patients.

This educational project revealed that, it is worth investing in educational programs involving nurses in the future. Supporting this conclusion are the results of a previous study by Xie et al. (2023), who showed that nurses’ knowledge of inhalation therapy is low. However, they pointed out that nurses working in community hospitals are better informed about inhalation therapy than those working in high-level hospitals, where the personnel have a higher workload. Giner et al. (2016) also observed that knowledge regarding using inhalers among nurses is poor, even despite recent training activities.

The rationale for conducting this educational project was evidence that many healthcare personnel still lack the basic knowledge and often technical skills necessary to teach patients inhalation techniques (Alismail et al., 2016). Medical personnel also rarely receive formal training in proper inhalation techniques (Bosnic-Anticevich, 2017), leading to an estimated 39%–67% of nurses, physicians, and respiratory therapists not adequately trained in patient education (Karle et al., 2020). Price et al. (2013) reported that only 15%–69% of healthcare personnel use inhalers correctly. A systematic review found that the inhalation technique among healthcare personnel has deteriorated recently. Additionally, it was observed that the vast majority did not use MDI and DPI inhalers correctly (Plaza et al., 2018).

The importance of the method of transmitting knowledge to the patient is also emphasized. It is believed that the most effective technique for educating on the correct use of an inhaler is verbal instruction combined with physical demonstration (“teach-back” technique) (Price et al., 2013). This type of education was implemented in our project, and the improvement in patient outcomes after education confirms that it is an effective method. Our project showed that after education in all study groups, patients achieved similar high results in improving their inhalation technique. Our observations are consistent with the opinion that when used correctly, there is little difference in clinical effectiveness between different inhalers (Bivolaru et al., 2023).

It has been shown that an incorrect inhalation technique, may be associated with poor asthma control (Lavorini and Usmani, 2013). Moreover, clinical experience shows that asthma medications are less effective in everyday practice than during a clinical trial (Román-Rodríguez et al., 2019; Lavorini and Usmani, 2013). Accordingly, our real-life study reflects everyday clinical practice conditions (Saturni et al., 2014). Randomization was not performed, and patients were selected based on the physician’s decisions during everyday practice. The lack of randomization may be a limitation due to the possibility of confounding variables and selection bias. On the other hand, the population in our study is heterogeneous and unselected, including patients with different medical histories, ages, genders, poor medication adherence, and poor use of the inhaler or inhalation technique. Therefore, the study can include all the variables that can influence outcomes in the conditions of daily medical practice. The results obtained in our study relate directly to typical patients of a health center and show their unique features. Therefore, practitioners can directly use the conclusions drawn in this educational project. It should be emphasized that patients were treated in the respiratory rehabilitation ward - these are specific conditions because patients repeat visits many times at different intervals. Therefore, the long-term follow-up was not planned, which may be considered as a limitation. Due to the pilot nature of the study, short-term effects were assessed, but it was not verified whether they were maintained over time. However, literature data indicate that although a single training session reduces errors in inhalation technique, its effects are not long-lasting, and the effects of a single training session are temporary and usually last up to 6 months after training (Dabrowska et al., 2019). This means that each training session requires systematic repetitions, and an additional, extended observation period may be introduced in future studies to assess the maintenance of inhalation skills over time. Another limitation related to the real-life nature is the lack of information from patients who had previously undergone training in the inhalation technique and how much time had passed since the last training. The study design did not assume collecting such information because, in the conditions in which the study was conducted, it was impossible to allocate patients to groups in terms of the type of inhaler and additional variables due to the limited number of participants. The limitation of the study is that there is also a small number of patients in the Ellipta and Turbuhaler groups. The small number of patients in these two groups results from the natural limitations of the study. This study was conducted in real-life conditions during everyday medical practice without a pre-imposed protocol. The number of patients in each group depended on how many patients using a specific type of inhaler were in the ward at the time of the study. We did not have the opportunity to extend the duration of the study and include more patients. However, these results should be interpreted with caution.

In conclusion, before education, the least accessible inhaler for patients was Ellipta, and the easiest to use was Cyclohaler. An important observation was that before education, the level of inhalation skills varied depending on the type of inhaler. However, education resulted in equalizing the level of correctness of inhalation, which turned out to be similarly high for each type of device. We have shown that adequately conducted education contributes to improving the inhalation technique of asthma patients. However, achieved improvement may be influenced by other factors such as practice effects or confounding variables due to real-life nature of the study. It should be emphasized that healthcare personnel play an essential role in developing the correct inhalation technique. The results indicate that a high level of education for nurses and patients was achieved. The study provides information on the importance of proper training of medical personnel. It also shows how effective nurse education improves patient therapy quality. Correct use of inhalers is crucial for the effectiveness of asthma treatment and symptom control, which can be achieved by training nurses, who will then pass on this knowledge to patients. As a result, patients will achieve better control of the disease, which will reduce the number of hospitalizations and medical interventions. Thanks to the education of nurses, patients will become more independent in managing their disease, which has reduced the need for frequent medical consultations. Such a model of cooperation between nurses and physicians contributes to a more efficient use of healthcare resources. This study is a starting point for further research on the role of nursing education in improving the quality of care for patients with chronic diseases, especially in inhaler use techniques. This study is a pilot study and may be the basis for developing new educational programs. The study’s conclusions indicate that investing in nurse education can not only improve treatment outcomes but will also contribute to the optimization of the healthcare system, which is of great importance in both the practical and scientific context.

## Data Availability

The original contributions presented in the study are included in the article/Supplementary Material, further inquiries can be directed to the corresponding author.
