# Supplementary material for: Effective inhaler technique education is achievable - assessment and comparison of five inhaler devices errors
Source: Front Pharmacol. 2025 Apr 24;16:1538283. doi: 10.3389/fphar.2025.1538283 (PMC12058497; doi:10.3389/fphar.2025.1538283)
Supplement: Supplementary file 1 [file DataSheet1.docx]

Supplementary Material

# Supplementary Table 1. Items subject to inhalation technique assessment.

| **Ellipta** | 1. Opening the inhaler cover  2. Slide the cover down until you hear a "click"^*^  3. Check the dose counter after hearing a “click”  4. Do not shake the inhaler before use (if the patient shakes the inhaler, it is an error)^**^  5. Exhale calmly and deeply  6. Exhale outside the inhaler  7. Putting the inhaler mouthpiece into your mouth and covering it tightly with your lips, without blocking it with your tongue  8. Blocking the air inlet with fingers^*^  9. Tilt your head slightly back  10. Inhaling as deeply and as intensely as possible through the mouth  11. Remove the inhaler from your mouth and hold your breath for at least 10 seconds  12. Exhale slowly out of the inhaler  13. Clean the inhaler mouthpiece with a dry wipe before closing the lid  14. Slide the lid up to cover the mouthpiece (two clicks audible) |
| --- | --- |
| **Diskus** | 1. Open the inhaler by sliding the cover until it stops and you hear a “click”^*^  2. Move the slider as far as it will go "away from you" until you hear a characteristic sound^*^  3. Do not shake the inhaler before use (if the patient shakes the inhaler, it is an error)^**^  4. Exhale calmly and deeply  5. Exhale outside the inhaler  6. Place the mouthpiece of the inhaler between your teeth and cover the mouthpiece tightly with your lips, without blocking it with your tongue  7. Tilt your head slightly back  8. Inhaling as deeply and as intensely as possible through the mouth  9. Remove the mouthpiece from your mouth and hold your breath for at least 10 seconds  10. Exhale slowly out of the inhaler  11. Close the inhaler by sliding the cover towards you until you hear a click |
| **Cyclohaler** | 1. Removing the cap  2. Hold the base of the inhaler firmly and open it by turning the mouthpiece in the direction of the arrow  3. The capsule is removed from the foil blister immediately before use  4. Placing the capsule in the capsule-shaped compartment at the base of the inhaler^*^  5. Turning the mouthpiece to the closed position  6. Press the colored buttons all the way down only once while holding the inhaler in a vertical position, then release the buttons^*^  7. Do not shake the inhaler before use (if the patient shakes the inhaler, it is an error)^**^  8. Exhale calmly and deeply  9. Exhale outside the inhaler  10. Putting the inhaler mouthpiece into your mouth and covering it tightly with your lips, without blocking it with your tongue  11. Tilt your head slightly back  12. Inhaling as deeply and as intensely as possible through the mouth  13. Making sure that the characteristic sound (whirring sound) is audible  14. Remove the inhaler from your mouth and hold your breath for at least 10 seconds  15. Exhale slowly out of the inhaler  16. Opening the inhaler and checking to see if there is any powder left in the capsule  17. Inhalation again if the medicine is left in the capsule  18. Removal of the empty capsule  19. Close the mouthpiece and put the cap back on |
| **pMDI** | 1. Remove the protective cap from the inhaler mouthpiece^*^  2. Take the inhaler between your index finger (possibly also middle finger) and thumb, holding it with the tip down  3. Shake the inhaler up and down several times  4. Release 1-4 sprays before using the inhaler for the first time or when the inhaler has been unused for > 4-7 days  5. Stand upright with your head slightly tilted back  6. Exhale slowly, as far as possible, out of the inhaler  7. Put the inhaler into your mouth, tightly covering the mouthpiece with your lips, and remove your tongue from the path of the aerosol  8. Press the canister and take a slow, deep breath in sync with the press (flow approx. 30-60 l/min, 2-4 s)^*^  9. Remove the inhaler from your mouth  10. Hold your breath for at least 10 seconds  11. Exhale slowly out of the inhaler for 2-4 seconds  12. Perform another inhalation (if necessary) after 15-60 seconds |
| **Turbuhaler** | 1. Unscrewing and removing the inhaler cap  2. Do not shake the inhaler before use (if the patient shakes the inhaler, it is an error)^**^  3. Hold the inhaler vertically with the colored dial facing down  4. Turn the red dial in one direction until it stops^*^  5. Then turn the inhaler in the opposite direction as far as it will go until you hear a characteristic sound (click)^*^  6. Exhale calmly and deeply  7. Exhale outside the inhaler  8. Place the mouthpiece of the inhaler between your teeth and cover the mouthpiece tightly with your lips, without blocking it with your tongue  9. Tilt your head slightly back  10. Inhaling as deeply and as intensely as possible through the mouth  11. Remove the mouthpiece from your mouth and hold your breath for at least 10 seconds  12. Taking the inhaler out of your mouth  13. Exhale slowly out of the inhaler  14. Place the cap on the inhaler and close it tightly |

^*^Critical error

^**^ Shaking the inhaler before use was considered an error because the type of inhaler used in the study (Ellipta, Diskus, Cyclohaler, Turbuhaler) did not require shaking before use. Only the pMDI used in the study required shaking before use (the study did not use pMDIs that did not require shaking before use).

**Supplementary Table 2. Summary of results.**

|  | **Ellipta** | **Diskus** | **Cyclohaler** | **pMDI** | **Turbuhaler** |
| --- | --- | --- | --- | --- | --- |
| **The most**  **common**  **mistake** | The patient shaked the inhaler before use despite this is not recommended^.*^  N=7 (100%) | The patient shaked the inhaler before use despite this is not recommended^*^  N=10 (83.33%) | The patient shaked the inhaler before use despite this is not recommended^*^  N=21 (70%) | The patient did not exhale as far as possible, slowly, out of the inhaler  N=16 (48.48%) | The patient shaked the inhaler before use despite this is not recommended^*^  N= 4  (57,14%) |
| **Average score**  **improvement**  **at 1^st^ visit** | 23% | 19% | 18% | 21% | 22% |
| **Average**  **difference in**  **score between**  **the 1^st^ and 2^nd^**  **visits** | 15% | 7% | 2% | 1% | 1% |
| **Average score**  **before**  **education** | 55% | 70% | 76% | 76% | 73% |
| **Average result after 1^st^ visit** | 79% | 89% | 94% | 97% | 96% |
| **Average score after education** | 94% | 96% | 96% | 99% | 97% |

Abbreviations: pMDI: pressurized metered-dose inhaler

^*^ Shaking the inhaler before use was considered an error because the type of inhaler used in the study (Ellipta, Diskus, Cyclohaler, Turbuhaler) did not require shaking before use. Only the pMDI used in the study required shaking before use (the study did not use pMDIs that did not require shaking before use).

**Supplementary Table 3. The differences between the number of critical errors and other errors.**

| **Chi-square test** | | | | | |
| --- | --- | --- | --- | --- | --- |
|  | **Ellipta** | **Discus** | **Cyclohaler** | **pMDI** | **Turbuhaler** |
| **Critical error I^*^**  **Before education to Visit 1, n *(p)*** | BE: 5  V1: 5  *(>0.1)* | BE: 2  V1: 0  *(0.14)* | BE: 5  V1: 0  *(****0.02)*** | BE: 8  V1: 4  *(0.2)* | BE: 1  V1: 0  *(0.3)* |
| **Critical error I^*^**  **Before education to Visit 2, n *(p)*** | BE: 5  V2: 1  *(0.51)* | BE: 2  V2: 0  *(0.14)* | BE: 5  V2: 0  ***(0.02)*** | BE: 8  V2: 1  ***(0.04)*** | BE: 1  V2: 0  *(0.3)* |
| **Critical error II^**^**  **Before education to Visit 1, n *(p)*** | BE: 2  V1: 0  *(0.13)* | BE: 1  V1: 0  *(0.3)* | BE: 1  V1: 0  *(0.31)* | BE: 0  V1:0  (-^***^) | BE: 1  V1: 0  *(0.3)* |
| **Critical error II^**^**  **Before education to Visit 2, n *(p)*** | BE: 2  V2: 0  ***(0.005)*** | BE: 1  V2: 0  *(0.3)* | BE: 1  V2: 0  *(0.31)* | BE: 0  V1: 0  *(-^***^)* | BE: 1  V1: 0  *(0.3)* |
| **Other errors**  **Before education to Visit 1, n *(p)*** | BE: 37  V1: 16  ***(0.0005)*** | BE: 36  V1: 13  ***(0.0002)*** | BE: 128  V1: 34  ***(<0.0001)*** | BE: 87  V1: 7  ***(<0.0001)*** | BE: 24  V1: 4  ***(<0.0001)*** |
| **Other errors**  **Before education to Visit 2, n *(p)*** | BE: 37  V2: 5  ***(<0.0001)*** | BE: 36  V2: 5  ***(<0.0001)*** | BE: 128  V2: 22  ***(<0.0001)*** | BE: 87  V2: 8  ***(<0.0001)*** | BE: 24  V2: 3  ***(<0.0001)*** |

Abbreviations: BE: before education; V1: Visit 1; V2: Visit 2; pMDI: pressurized metered-dose inhaler

^*^ The critical error I: for Ellipta – Blocking the air inlet with fingers; for Diskus – Move the slider as far as it will go "away from you" until you hear a characteristic sound; for Cyclohaler – Press the colored buttons all the way down ONLY ONCE while holding the inhaler in a vertical position, then release the buttons; for Pressurized MDI – Press the canister and take a slow, deep breath in sync with the press (flow approx. 30-60 l/min, 2-4 s); for Turbuhaler – Turn the inhaler in the opposite direction as far as it will go until you hear a characteristic sound (click).

^**^ The critical error II: for Ellipta – Slide the cover down until you hear a "click"; for Diskus – Open the inhaler by sliding the cover until it stops and you hear a click; for Cyclohaler – Placing the capsule in the capsule-shaped compartment at the base of the inhaler; for Pressurized MDI – Remove the protective cap from the inhaler mouthpiece; for Turbuhaler – Turn the red dial in one direction until it stops.

^***^ For critical error II before education to Visit 1 and Visit 2 statistical significance was not calculated due to the absence of this type of error in the group at Visit 1 and Visit 2 and before education.


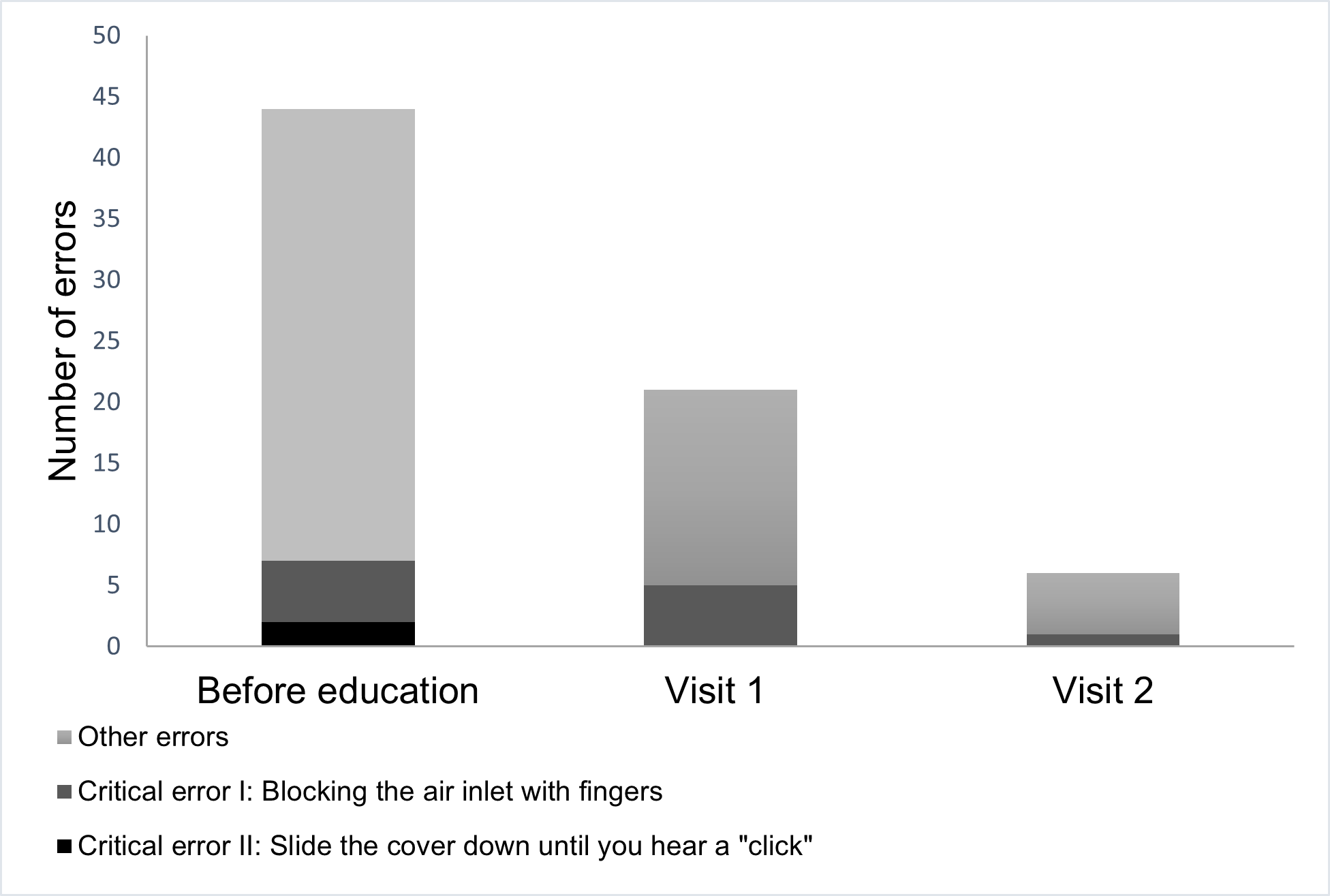


**Supplementary Figure 1.** **Number of patients’ errors for over the subsequent study visits for Ellipta.**

Proportional distribution of errors considered critical when using the inhaler in relation to other errors.

Calculations for this part of the analysis and cumulative charts were made in a spreadsheet as an additional graphic element to illustrate differences in the number of errors. This part was not subject to statistical analysis.


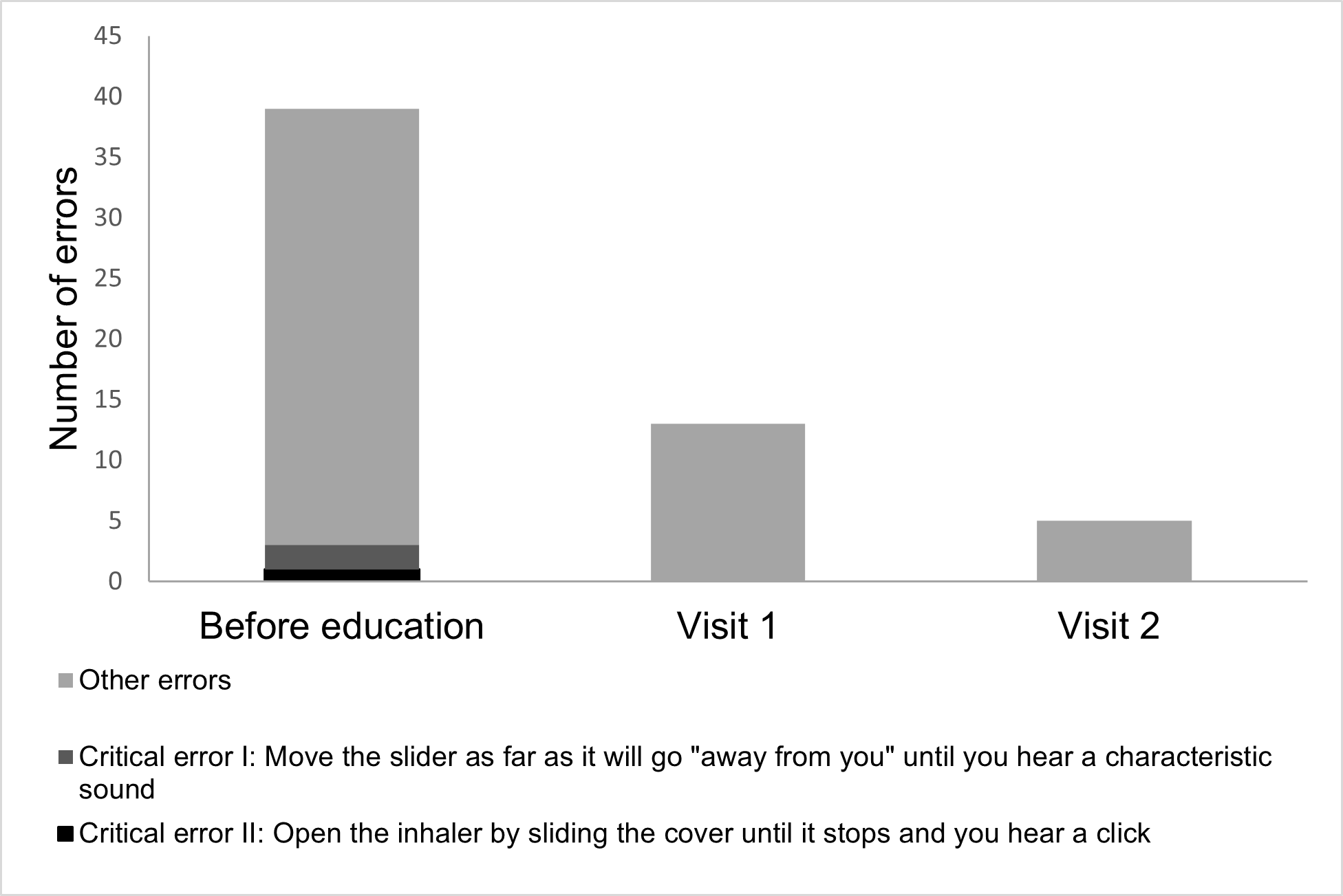


**Supplementary Figure 2.** **Number of patients’ errors for over the subsequent study visits for Diskus.**

Proportional distribution of errors considered critical when using the inhaler in relation to other errors.

Calculations for this part of the analysis and cumulative charts were made in a spreadsheet as an additional graphic element to illustrate differences in the number of errors. This part was not subject to statistical analysis.


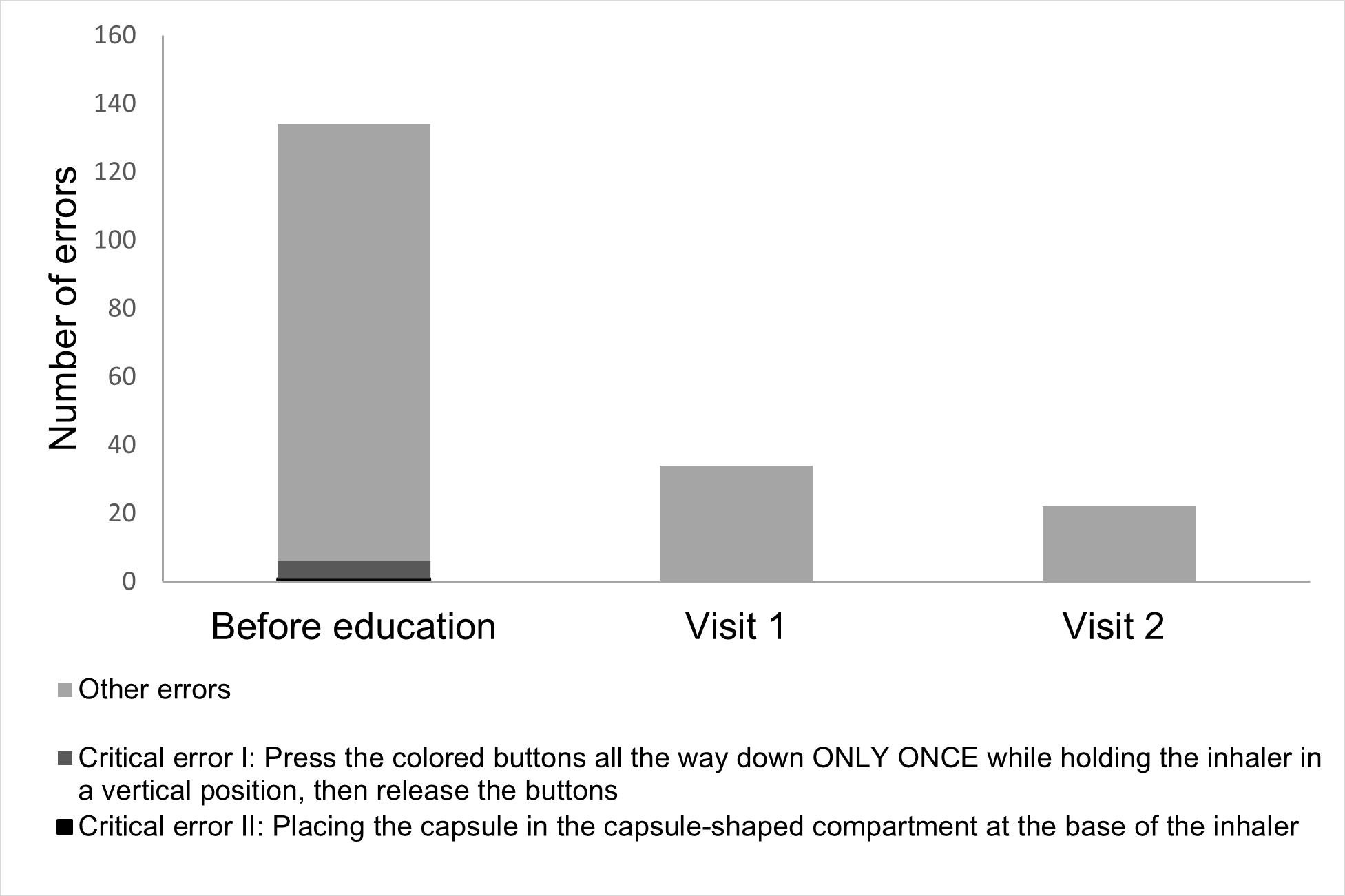


**Supplementary Figure 3.** **Number of patients’ errors for over the subsequent study visits for Cyclohaler.**

Proportional distribution of errors considered critical when using the inhaler in relation to other errors.

Calculations for this part of the analysis and cumulative charts were made in a spreadsheet as an additional graphic element to illustrate differences in the number of errors. This part was not subject to statistical analysis.


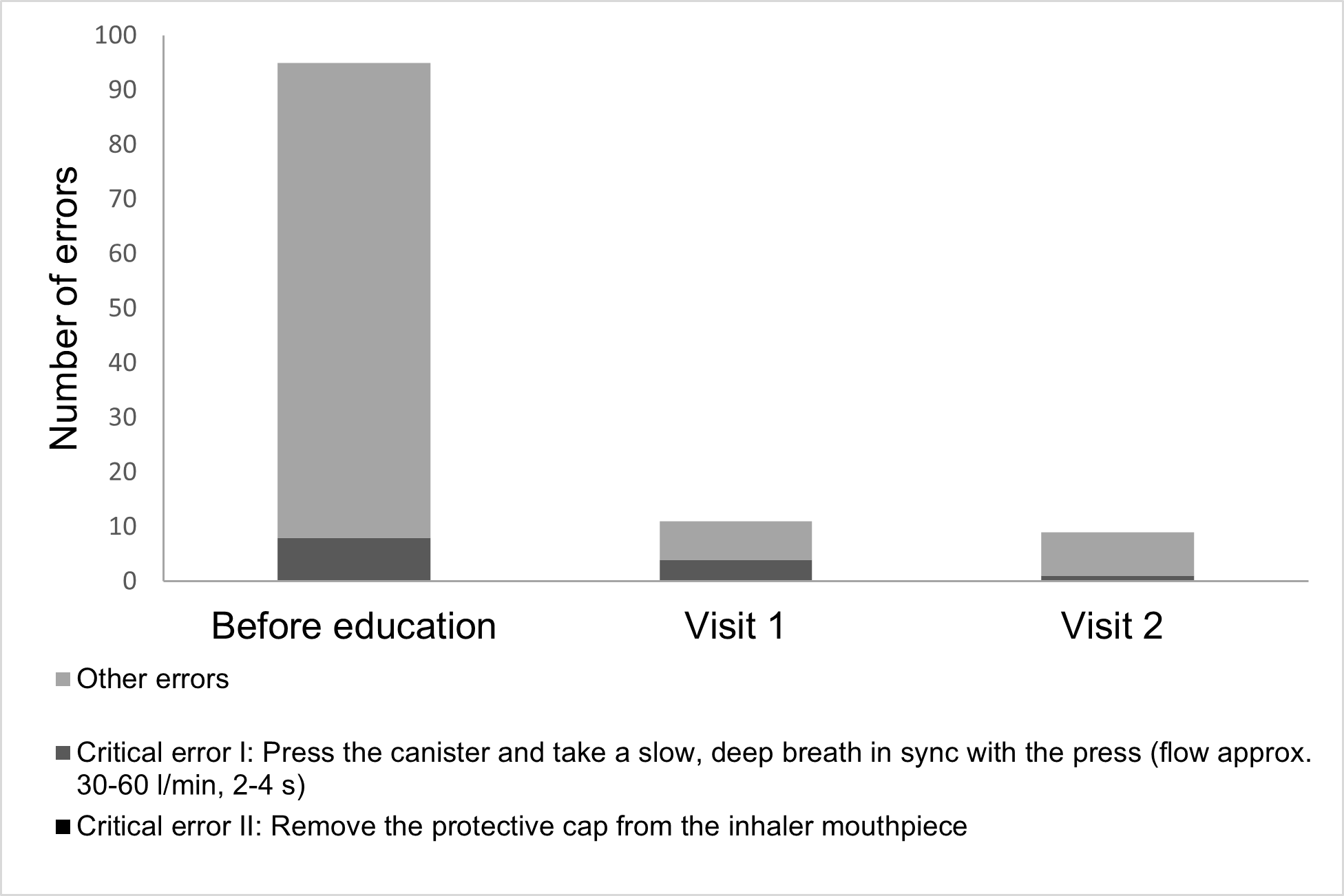


**Supplementary Figure 4.** **Number of patients’ errors for over the subsequent study visits for Pressurized MDI.**

Proportional distribution of errors considered critical when using the inhaler in relation to other errors.

Calculations for this part of the analysis and cumulative charts were made in a spreadsheet as an additional graphic element to illustrate differences in the number of errors. This part was not subject to statistical analysis.


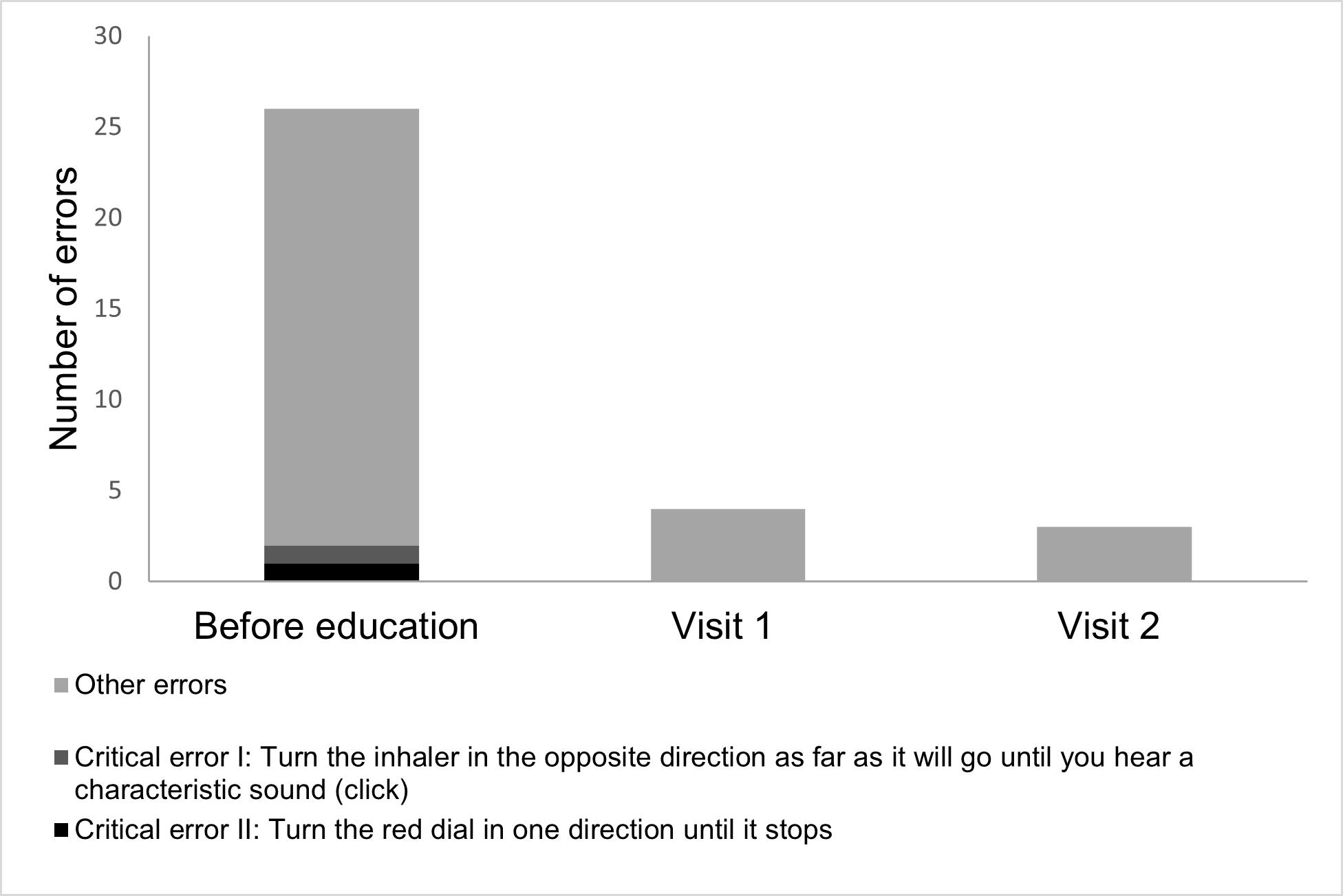


**Supplementary Figure 5.** **Number of patients’ errors for over the subsequent study visits for Turbuhaler.**

Proportional distribution of errors considered critical when using the inhaler in relation to other errors.

Calculations for this part of the analysis and cumulative charts were made in a spreadsheet as an additional graphic element to illustrate differences in the number of errors. This part was not subject to statistical analysis.
